# Decrypting Integrins by Mixed-Solvent Molecular Dynamics Simulations

**DOI:** 10.1021/acs.jcim.3c00480

**Published:** 2023-06-13

**Authors:** Ioana M. Ilie, Claus Ehrhardt, Amedeo Caflisch, Gabriele Weitz-Schmidt

**Affiliations:** † van ’t Hoff Institute for Molecular Sciences, University of Amsterdam, P.O. Box 94157, 1090 GD Amsterdam, The Netherlands; ‡ Amsterdam Center for Multiscale Modeling (ACMM), University of Amsterdam, P.O. Box 94157, 1090 GD Amsterdam, The Netherlands; § Department of Biochemistry, University of Zurich, Winterthurerstrasse 190, CH-8057 Zurich, Switzerland; ∥ AlloCyte Pharmaceuticals AG, Hochbergerstrasse 60C, CH-4057 Basel, Switzerland

## Abstract

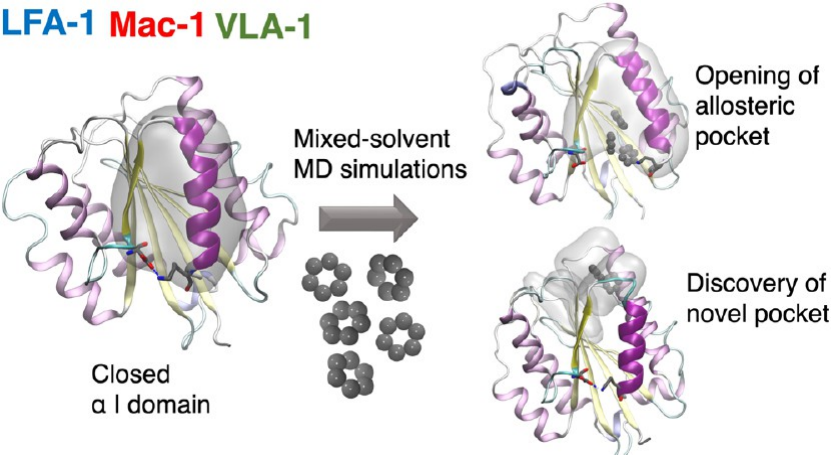

Integrins are a family of α/β heterodimeric cell surface adhesion receptors which are capable of transmitting signals bidirectionally across membranes. They are known for their therapeutic potential in a wide range of diseases. However, the development of integrin-targeting medications has been impacted by unexpected downstream effects including unwanted agonist-like effects. Allosteric modulation of integrins is a promising approach to potentially overcome these limitations. Applying mixed-solvent molecular dynamics (MD) simulations to integrins, the current study uncovers hitherto unknown allosteric sites within the integrin α I domains of LFA-1 (α_L_β_2_; CD11a/CD18), VLA-1 (α_1_β_1_; CD49a/CD29), and Mac-1 (α_M_β_2_, CD11b/CD18). We show that these pockets are putatively accessible to small-molecule modulators. The findings reported here may provide opportunities for the design of novel allosteric integrin inhibitors lacking the unwanted agonism observed with earlier as well as current integrin-targeting drugs.

## Introduction

Integrins are a 24-membered family of α/β heterodimeric cell surface receptors which are expressed in cell lineage defined arrays throughout the organism. They mediate cell adhesion, migration, differentiation, and proliferation. The activity of integrins is regulated by coordinated global conformational rearrangements. Signals from inside the cells convert integrins from their inactive bent conformation to their active, extended conformation, which allows ligands to bind (inside-out signaling). Conversely, ligand binding stabilizes the active integrin state and conveys signals back into the cell (outside-in signaling).^1,2^ This bidirectional signaling capability is unique to integrins and enables cells to dynamically respond to microenvironmental changes.^3^

**Figure 1:**
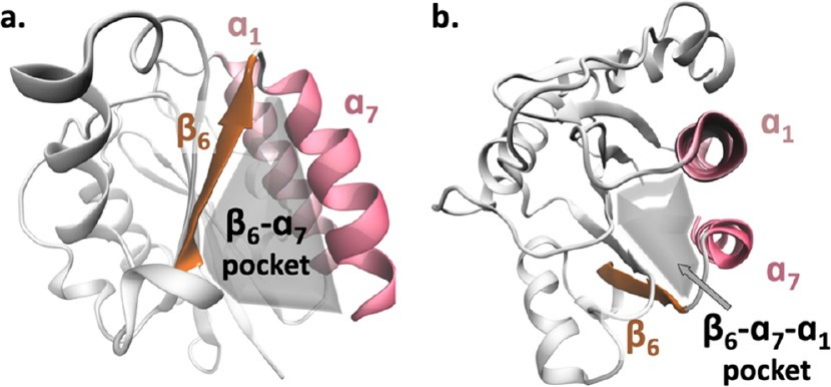
Ribbon representation of the α I domain of LFA-1 (PDB ID: 2ICA(5)) in (a) side view and (b) top view. Highlighted are the β_6_ β-strand (orange), helices α_1_ (pink) and α_7_ (red). The classical α I allosteric site (herein also referred to as β_6_–α_7_ pocket) and the newly identified β_6_–α_7_–α_1_ pocket are shown in gray.

Integrins can be categorized into two major subfamilies based on the presence or absence of a globular domain inserted in the top part of the integrin α subunit, termed α I domain. Nine out of the 18 known integrin α chains contain an α I domain (Figure 1). If present, this domain serves as the primary binding domain for integrin ligands. Located at the upper surface of the α I domain a single divalent cation-binding site, the so-called MIDAS (metal ion-dependent adhesion site) has been demonstrated to directly interact with integrin ligands. At the distal bottom face, the C-terminal α_7_ helix of the α I domain is central to integrin affinity regulation. A downward axial displacement of the α_7_ helix determines the transition of the α I domain from a low-affinity state to a high-affinity state, thereby enhancing its ligand binding affinity by up to 10,000-fold.^4^

Integrins play crucial roles in diseases of high unmet medical need including cardiovascular disease, thrombosis, inflammation, and cancer, rendering them attractive therapeutic targets.^6−8^ However, the development of integrin-targeting medications over the past decades found itself confronted with substantial challenges reiteratively defeating therapeutic expectations.^8−10^ A particularly profound challenge with integrin inhibition is the potential of unwanted agonist-like effects (i.e., effects opposite to the intended effects). Such unwanted partial agonism has been mostly observed with ligand mimetics interacting with the native ligand binding site of the integrin. The unintended triggering of these agonist-like effects is considered a main reason for reiterative late clinical-stage development failures of integrin-targeting interventions.^8,9,11^

Allosteric integrin modulation stabilizing integrins in the desired affinity state holds the promise of resolving this pharmacological challenge.  The feasibility of allosteric integrin modulation by small molecules was demonstrated early for the α I domain-containing integrin lymphocyte function-associated antigen-1 (LFA-1, α_L_β_2_, CD11a/CD18).^12,13^ The respective allosteric site (termed α I allosteric site or lovastatin (L)-site) has been discovered by serendipity. It resides within the α I domain of LFA-1 and is located underneath the C-terminal α_7_ helix of the α I domain (Figure 1). Small molecules binding to this site inhibit LFA-1 function by locking LFA-1 in the inactive bent conformation.^4^ To date, chemically diverse small-molecule LFA-1 inhibitors have been described, which are active in the low nanomolar range in vitro assay systems and efficacious in vivo in experimental disease models.^14−17^ Moreover, as predicted from their mechanism of action, these allosteric inhibitors do not induce unwanted agonist-like effects observed with previous integrin-targeting modalities. Further, they display high selectivity for LFA-1 versus other integrins, differentiating them from most orthosterically acting integrin inhibitors.^17−19^

Setting out for the current study, we hypothesized that α I allosteric sites similar to the LFA-1 α I allosteric site may be accessible in other α I domain-containing integrins. For the identification of such therapeutically employable allosteric sites, several computational and experimental methods are available to date.^20^ For our study, we employ mixed-solvent molecular dynamics (MD) simulations, which use small probes (mostly hydrophobic organic molecules) mixed with water to detect cryptic sites in proteins. The application of this method has several advantages: (1) it allows for protein flexibility, (2) it takes competition between the probe and water into account, and (3) it is validated.^21−25^ In particular, a novel cryptic pocket with druggable properties was identified in the SARS-CoV-2 spike glycoprotein via mixed-solvent MD simulations and validated via hydrogen–deuterium exchange mass spectrometry experiments.^23^ When combined with virtual screening, better performance in docking to ensembles extracted from mixed-solvent simulations was seen.^21^ The druggability of the major pocket of the nonstructural protein 1 of SARS-CoV-2 (cryptic) was recently demonstrated by mixed-solvent MD simulations and validated by fragment-based screening via X-ray crystallography,^24^ further strengthening the potential of the approach.

**Figure 2:**
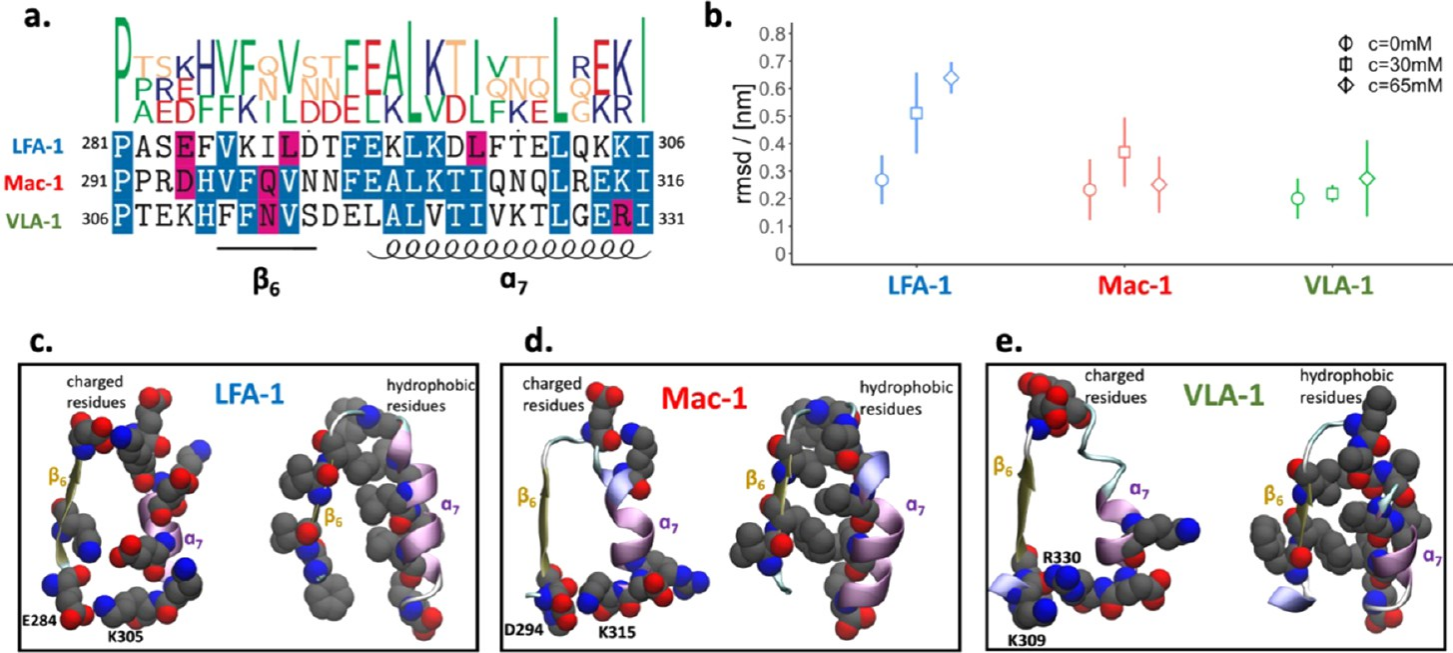
Characteristics of the α I domain β_6_–α_7_ interface. (a) Alignment of the β_6_–α_7_ sequences across the analyzed α I domains. The pink, blue, and white backgrounds indicate residue similarity, conservation more than 50%, and no conservation, respectively, across the three systems. (b) Average root-mean-square deviations (RMSD) of α_7_ with respect to the rest of the I domain. The reference structure is the closed conformation. For LFA-1, the reference structure is a representative closed conformation post-equilibration. First structural alignment of the individual snapshots saved along the MD simulations is carried out on the Cα-atoms of all residues except those comprised in α_7_, i.e., F292-I306, F302-I316, and E317-I331 for LFA-1, Mac-1, and VLA-1, respectively. Then for each MD snapshot, the α_7_ Cα-RMSD is calculated as , where *r*_*i*_ and *r*_*i*_^ref^ are the actual and reference coordinates, respectively, of the α_7_ Cα atom *i*, and *N* is the number of residues in α_7_. The error bars represent the standard error of the mean calculated as the standard deviation of the average values over the five independent runs. (c–e) Snapshots of the three systems focusing on the β_6_–α_7_ interface in the ribbon representation and highlighting the charged (left snapshot) or the hydrophobic residues (right snapshot) by van der Waals spheres.

As integrin model systems for the mixed-solvent simulations, we chose the α I domains of the integrins LFA-1, macrophage-1 antigen (Mac-1, α_M_β_2_, CD11b/CD18), and very late antigen-1 (VLA-1, α_1_β_1_, CD49a/CD29). All three integrins have been associated with autoimmune and malignant diseases.^26,27^ Thus, the search for novel, potentially targetable allosteric sites within their α I domains holds substantive therapeutic promise. Their α I domains have been extensively characterized at molecular levels.^2,28,29^ Structurally, the three α I domains consist of six β-strands and seven α-helices which form a compact conformation (LFA-1: PDB ID: 2ICA;^5^ Mac-1: PDB ID:1NA5^30^ and VLA-1: PDB ID 1PT6(31)). The overall structure is conserved with some changes in the loops, particularly in the G283 to T292 loop of VLA-1, which is longer than the corresponding ones of LFA-1 and Mac-1. This loop is not in contact with β_6_–α_7_ segment, where inhibitors were previously identified for LFA-1.^5^ The sequence alignment focusing on the β_6_–α_7_ segment reveals partial conservation of the residues in the individual sequences (blue background in Figure 2a). Essentially, the previously identified allosteric pocket is stabilized by the E284-K305 salt bridge in LFA-1 and D294-K315 in Mac-1. The analogue contact in VLA-1 is K309-R330, which can form hydrogen bonds between the K309 backbone oxygen atom and the R330 side chain.

By applying mixed-solvent MD simulations to integrins, we report here the opening of α I allosteric sites analogous to the classical α I allosteric site of LFA-1 in the integrins Mac-1 and VLA-1. Moreover, the MD simulations identify a novel pocket in all three integrin α I domains studied (termed α_6_–β_7_–α_1_ pocket) shown to be accessible to small molecules by molecular docking studies. Potential implications of these findings for the design of next-generation integrin-targeting drugs are discussed.

## Results

### α I Allosteric Site Closes in the Absence of Inhibitors and Becomes Flexible in the Presence of Fragment Probes

To identify new α I allosteric pockets and to implicitly probe the effects of hydrophobic compounds on their structure and dynamics, we used the α I domains of the integrins LFA-1, Mac-1, and VLA-1 as model systems. We started from existing crystal structures of the selected α I domains (LFA-1: PDB ID: 2ICA;^5^ Mac-1: PDB ID:1NA5^30^ and VLA-1: PDB ID 1PT6(31)). We first removed the allosteric ligand co-crystallized with the LFA-1 α I domain. The crystal structures of the Mac-1 and VLA-1 α I domains did not contain any ligands (referred to as closed structures). Using these α I domains, we performed long molecular dynamics simulations in the presence and absence of fragments (benzene). In brief, six different sets of simulations were carried out for each system, using three different concentrations of benzene (0, 30, and 65 mM, respectively) and temperature values of 300 and 350 K. Five independent 1-μs simulations were carried out for each combination of benzene concentration and temperature for a cumulative sampling of 30 μs as described in the Methods section. In this report, we focus the analysis on the production simulations at 300 K. Similar results were obtained at 350 K with the main difference shown in the Supporting Information.

Upon removal of ligand and in the absence of fragments, the LFA-1 α I allosteric site between β_6_ and the C-terminal α_7_ helix closed within the first 5 ns of equilibration (not shown). This rapid closing is consistent with previously reported NMR solution phase structures of the LFA-1 α I domain. These structures reveal that the β_6_–α_7_ region where α I allosteric ligands bind to is occluded. In consequence, a segmental (i.e., rigid body) movement of the C-terminal α_7_ helix is mandatory to allow ligands to interact in solution.^32,33^ Additionally, we show here that in the absence of fragments, the average root-mean-square deviations (RMSDs) of α_7_ from the closed conformation of the LFA-1 α I allosteric site and the respective putative sites in Mac-1 and VLA-1 (β_6_–α_7_ pockets) (Figure 2b, empty circles) are small (below 2.7 ± 0.1 Å), indicating a preserved closed conformation (Movies S1–S3).

These simulation results motivated the use of mixed-solvent MD simulations to uncover putative α I allosteric pockets in Mac-1 and VLA-1. To prompt the opening of these pockets, the proteins were solvated in an aqueous mixture with benzene molecules at different concentrations. The choice of molecules was prompted by studies supporting the hypothesis of fragment-probe-induced pocket formation.^23^ The RMSD analysis revealed that increased benzene concentration leads to higher deviations from the closed conformation of the β_6_–α_7_ pocket, with the most pronounced effect observed for LFA-1 (Figure 2b). The effect was marginal for Mac-1 and VLA-1, which can be ascribed to the polarity of the residues at the β_6_–α_7_ interface. For LFA-1, charged residues interact with bulk water and contribute to a large extent to the flexibility of the pocket (Figure 2c, left). Hydrophobic residues are also present, but their side chains in α_7_ are far from apolar residues in β_6_ (Figure 2c, right). In contrast, the Mac-1 and VLA-1 interfaces are rich in hydrophobic residues pointing toward each other, contributing to their closed conformation (Figure 2d,e, right snapshots). In addition, the closed conformation is also stabilized by the D294-K315 salt bridge or the K309-R330 contact in Mac-1 and VLA-1, respectively.

Higher temperatures lead to increased deviations for Mac-1 and VLA-1 and comparable deviations for LFA-1 (Figure S1). For the three integrin α I domains, the high error bars indicate more frequent transitions from open to closed conformations of the pocket.

**Figure 3:**
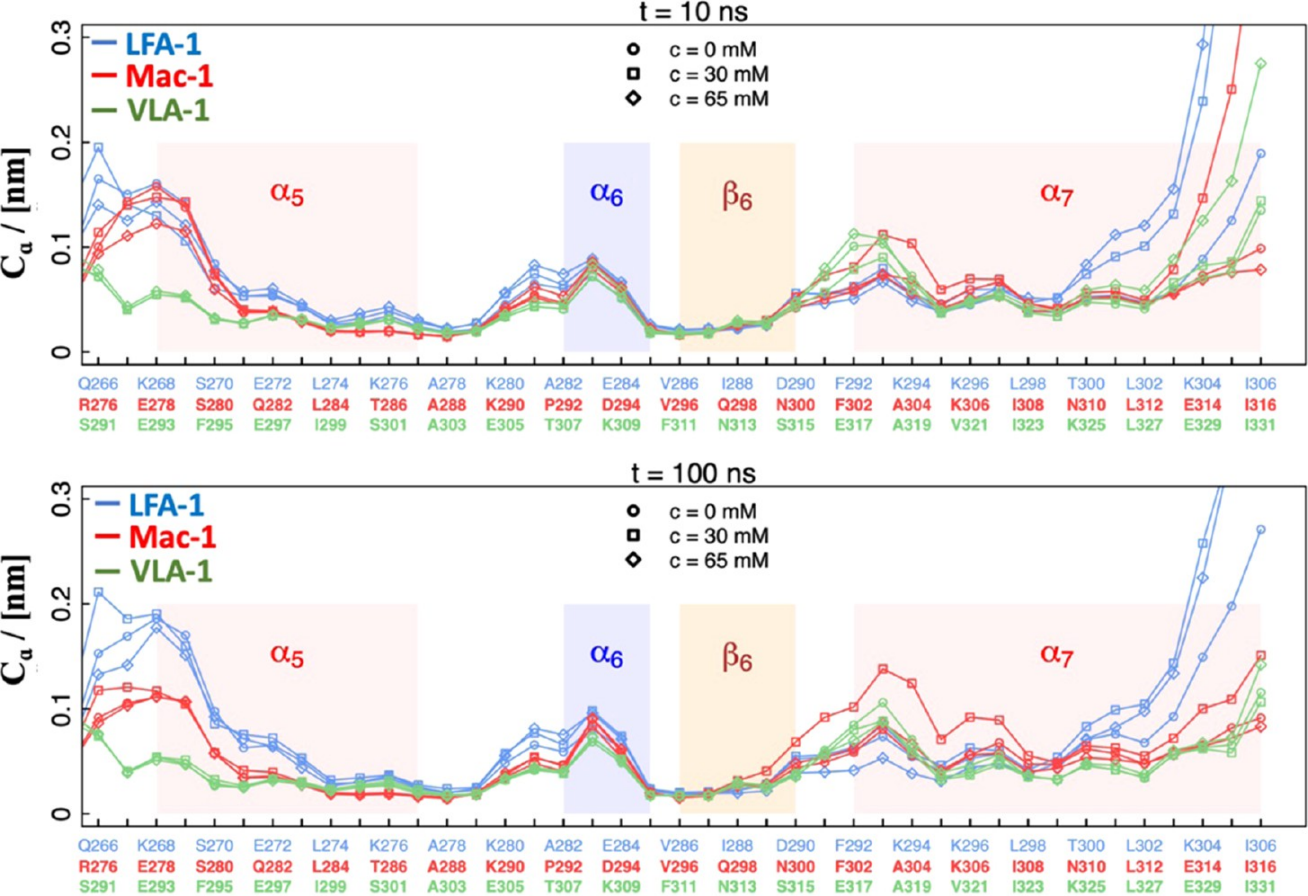
RMSF profiles of the α I domains. Two different timescales are shown comprising fluctuations over 10 and 100 ns. The RMSF profiles were calculated as the average over 500 independent 10-ns and 50 independent 100-ns profiles, for the short and the long timescales, respectively. The fluctuations were calculated about the average structure determined from the corresponding 10- or 100-ns intervals.

The analysis, focusing on the flexibility of the α_7_ helix of the α I domains on two different timescales (10 and 100 ns, Figure 3, top and bottom, respectively) revealed that the C-terminus of α_7_ is more susceptible to perturbations induced by the probe molecules on the short timescale than on the long timescale, while the plasticity of other segments is similar at both timescales. Independently of the analyzed system, the profiles show the least fluctuations in the last eight residues of α_7_ in the absence of benzene molecules. Increasing the benzene concentration leads to higher fluctuations of the C-terminus of α_7_ in LFA-1. This effect was more pronounced on the shorter timescale. Interestingly, intermediate benzene concentrations (30 mM) increase the fluctuations of the N- and C-termini of α_7_ in Mac-1, and high concentrations have only a minor impact on the fluctuations compared to the system devoid of benzene. The C-terminus of VLA-1 shows higher plasticity only at high benzene concentration (65 mM) on the short timescale, while negligible differences in flexibility are observed as a function of benzene concentration on the long timescale. The more pronounced effects on the short timescale show the dynamic response of the α I domain to benzene, which is an indication of the opening and closing of the β_6_–α_7_ pocket.

### High Benzene Concentration Reduces the Population of Stabilizing Salt Bridges

**Figure 4:**
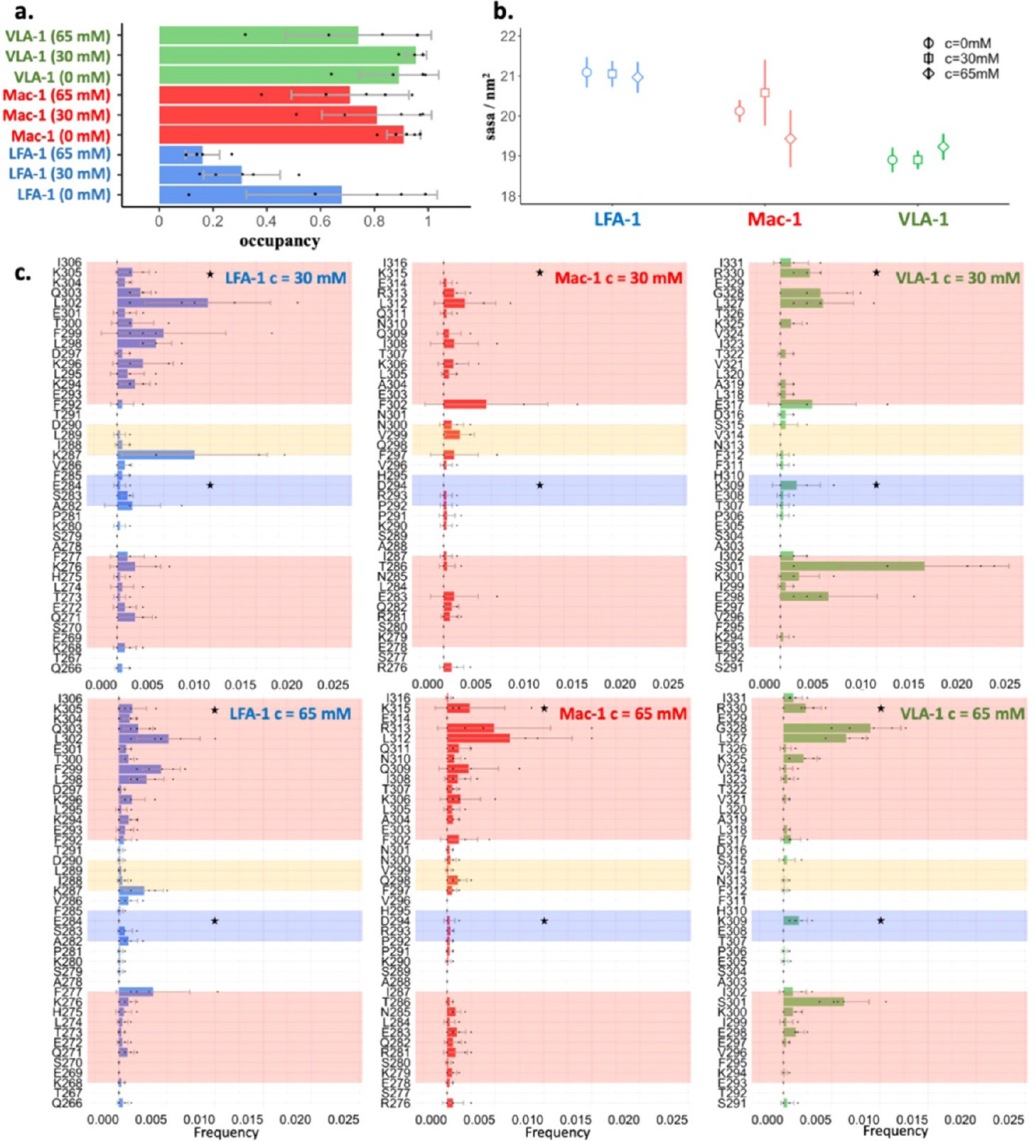
Effects of benzene interaction with α_7_. (a) Occupancy of the E284-K305, D294-K315 salt bridges, and the K309-R330 contact. A salt bridge is considered to form when the distance between the E284 C_δ_ and K305 N_ζ_ atoms and the distance between the D294 C_γ_ and the K315 N_ζ_ atoms is below 0.5 nm in LFA-1 and Mac-1, respectively. Analogously, a contact is preserved in VLA-1 when the distance between the backbone oxygen atom of K309 and the C_ζ_ of R330 is below 0.7 nm. (b) Solvent-accessible surface area of α_7_, i.e., the F292-I306, F302-I316, and E317-I331 segments for LFA-1, Mac-1, and VLA-1, respectively. (c) Interaction frequencies between the α I domains and the benzene molecules mapped on the sequence of the proteins and normalized by the number of benzenes in the system. A contact is formed between the benzene molecules and a residue if the distance between any of their atoms is below 0.5 nm. The error bars represent the standard error calculated as the standard deviation of the five independent values.

The E284-K305 salt bridge has been previously identified to play an important role in preserving the closed conformation of the β_6_–α_7_ pocket in LFA-1.^34^ The corresponding salt bridge in Mac-1 is D294-K315 and the analogue contact in VLA-1 is K309-R330, which can form hydrogen bonds (H-bonds) between the K309 backbone oxygen atom and the R330 side chain. Results show that these polar contacts are mostly populated in the absence of benzene molecules and are differently affected with increasing benzene concentration across the distinct α I domains (Figure 4a, Movies S1–S9). Visual inspection reveals a clear trend toward more pronounced pocket opening effects at 65 mM of benzene (Movies S7–S9) compared to a benzene concentration of 30 mM (Movies S4–S6). The LFA-1 salt bridge is the most prone to break, showing a decrease of occupancy of more than 60% at 65 mM compared to physiological conditions. Note that compared to the analogous salt bridge in Mac-1 and the H-bond in VLA-1, the LFA-1 salt bridge is less populated in the absence of benzene molecules. Adding benzene to the solvent has a comparable effect on the stabilizing contact in Mac-1 and VLA-1, with a decrease of about 30% in occupancy at 65 mM benzene. Interestingly, at intermediate benzene concentrations, VLA-1 is predominantly more stable than at high concentrations (yet remains within the error bar at 65 mM). The susceptibility of the LFA-1 α I allosteric site to open more easily is also reflected by the solvent-accessible surface area of its α_7_, which is the largest across the three systems independently of the benzene concentration (Figure 4b). The α_7_ of VLA-1 is the least solvent exposed and is only marginally influenced by the addition of benzene molecules, while for Mac-1 the α_7_ solvent exposure is reduced. The large error bars for Mac-1 are an indication of the variability of the system and correlate well with the flexibility of α_7_.

**Figure 5:**
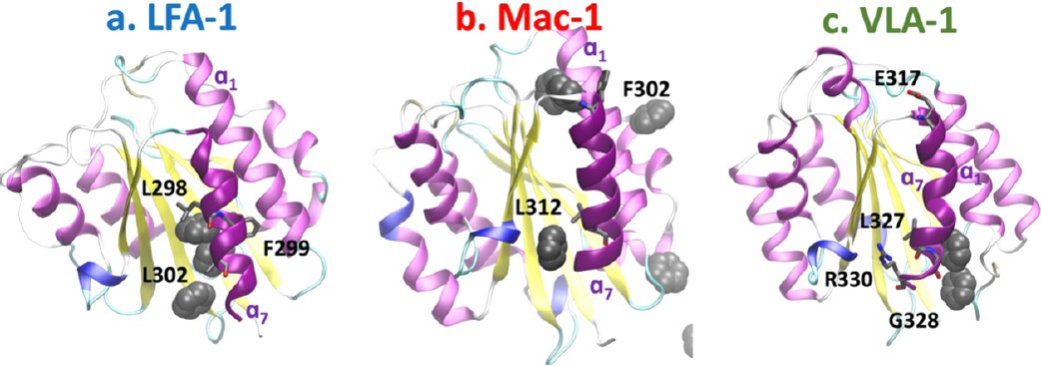
Interactions between the benzene molecules and the α I domains. α I domains (ribbons) of LFA-1 (a), Mac-1 (b), and VLA-1 (c). Highlighted are the residues (sticks) in α_7_ (dark purple helix) that interact with the benzene rings (gray van der Waals spheres).

The contact map analysis (Figure 4c) shows that the benzene molecules rarely interact with the residues involved in the salt bridge in LFA-1 and Mac-1 (black stars in Figure 4c) but rather engage with preponderance in nonpolar interactions with leucine and phenylalanine side chains. In contrast, in VLA-1 the benzene molecules interact with the alkyl (i.e., hydrophobic) moiety of the K309 and R330 side chains, though they also contact residues L327-G328. The disruptive effect of benzene on the stabilizing contact arises from the more frequent interactions of the molecules with the residues in the core of α_7_ and its surrounding. In practice, this translates in LFA-1 into the insertion of benzene molecules between α_7_ and β_6_, and α_7_ and α_1_, thereby pushing α_7_ away from β_6_ and opening the allosteric pockets. Secondary effects include the displacing of α_7_ away from α_1_ (Figure 5a, Movies S4 and S7), which could give rise to a new allosteric pocket. Similarly, in Mac-1 and VLA-1, the interactions of the benzene rings with L312 and F302, and L327 and G328, respectively, loosen the contacts between α_7_ and α_1_ (Figure 5b,c, Movies S8 and S9).

### SAPPHIRE Analysis Reveals a Novel Pocket

**Figure 6:**
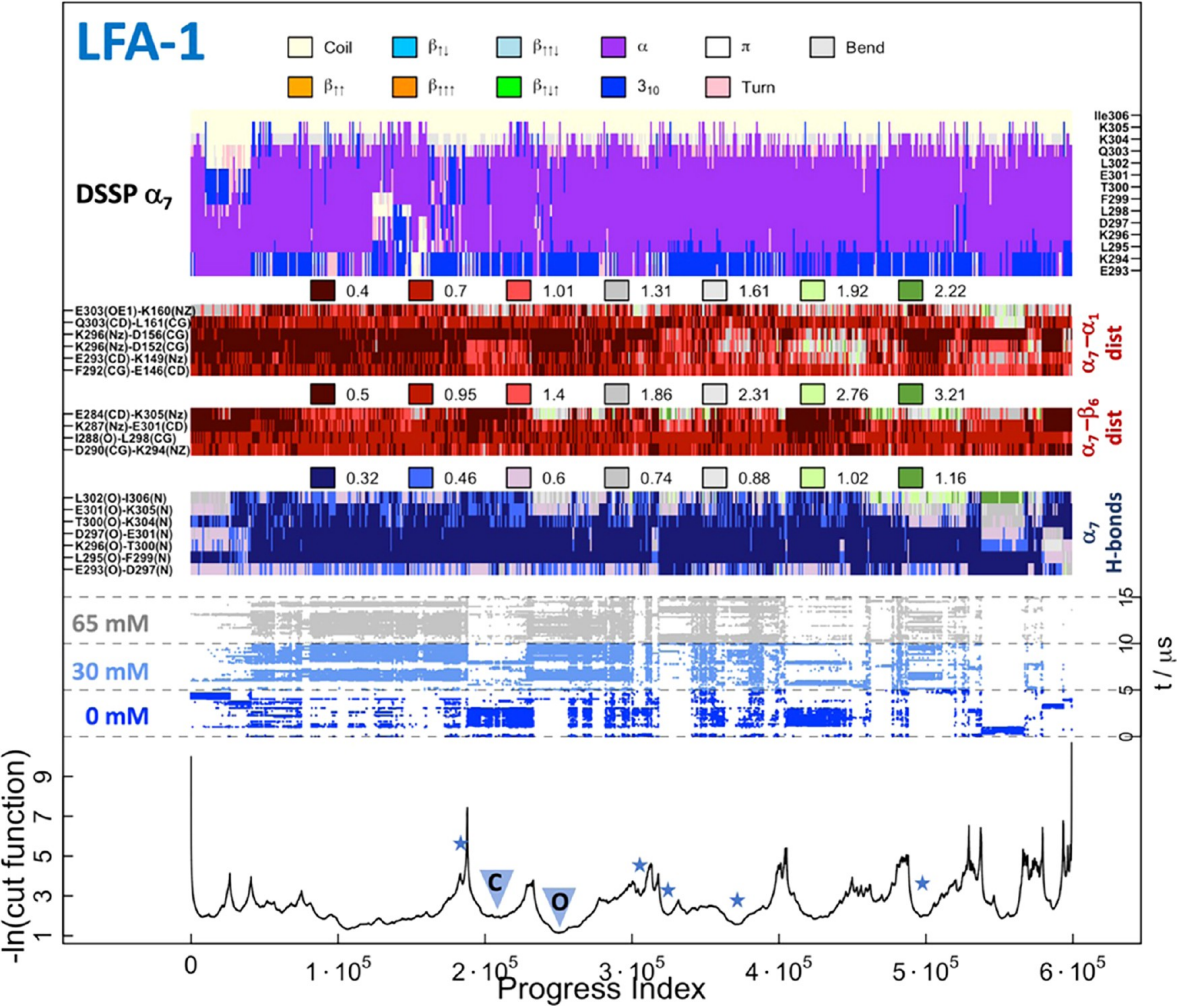
SAPPHIRE plot for the conformational space of the LFA-1 β_6_–α_7_ pocket. (Bottom) The progress index corresponds to the reordering of the snapshots according to pairwise structural similarity. The cut function (black line) is constructed by counting transitions along the simulations such that its local minima and maxima correspond to states that are highly populated and barriers that are visited sporadically, respectively. The blue stars mark the identified open conformations, and the triangles highlight the closed and open conformations discussed in the main text and shown in the figure. The dynamic trace (dark blue, light blue, and gray dots) localizes the time development of the simulated system along the progress index and cut function. In other words, the dynamic trace reflects the sequence of events as it illustrates the visits to individual states and crossing of barriers for each simulation run where individual systems are separated by horizontal dotted lines and highlighted by the different colors. (Middle) Intramolecular distances that contribute to the stability of the allosteric pocket, i.e., donor–acceptor α_7_-intramolecular distances (α_7_ H-bonds), α_7_–β_6_ distances (α_7_–β_6_ dist), and α_7_–α_1_ distances (α_7_–α_1_ dist). The complete list of distances used for the progress index metric is in Table S1. Each set of distances has values in nm grouped into seven bins (color legend on the top of each set of distances). (Top) Secondary structure assignment of α_7_ with its corresponding legend on top.

**Figure 7:**
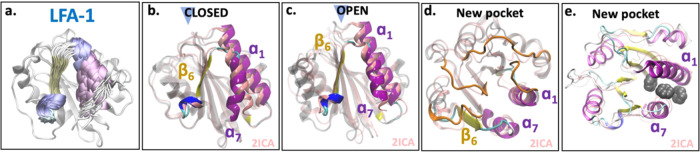
Simulation snapshots of LFA-1. (a) Structural overlap of 60 snapshots extracted from the simulations highlighting the dynamics of α_7_. (b) Representative snapshots of the closed and (c) open conformations of the allosteric pocket extracted from the center of the basins highlighted in the SAPPHIRE plot (Figure 6). The structural overlap to the open LFA-1 crystal structure (PDB ID: 2ica(5)) is shown. Highlighted are β_6_, α_7_, and α_1_. (d, e) Top view of the new pocket, highlighting the loops (orange loops in (d)) and the insertion of benzene molecules (shown in van der Waals representation in (e)).

**Figure 8:**
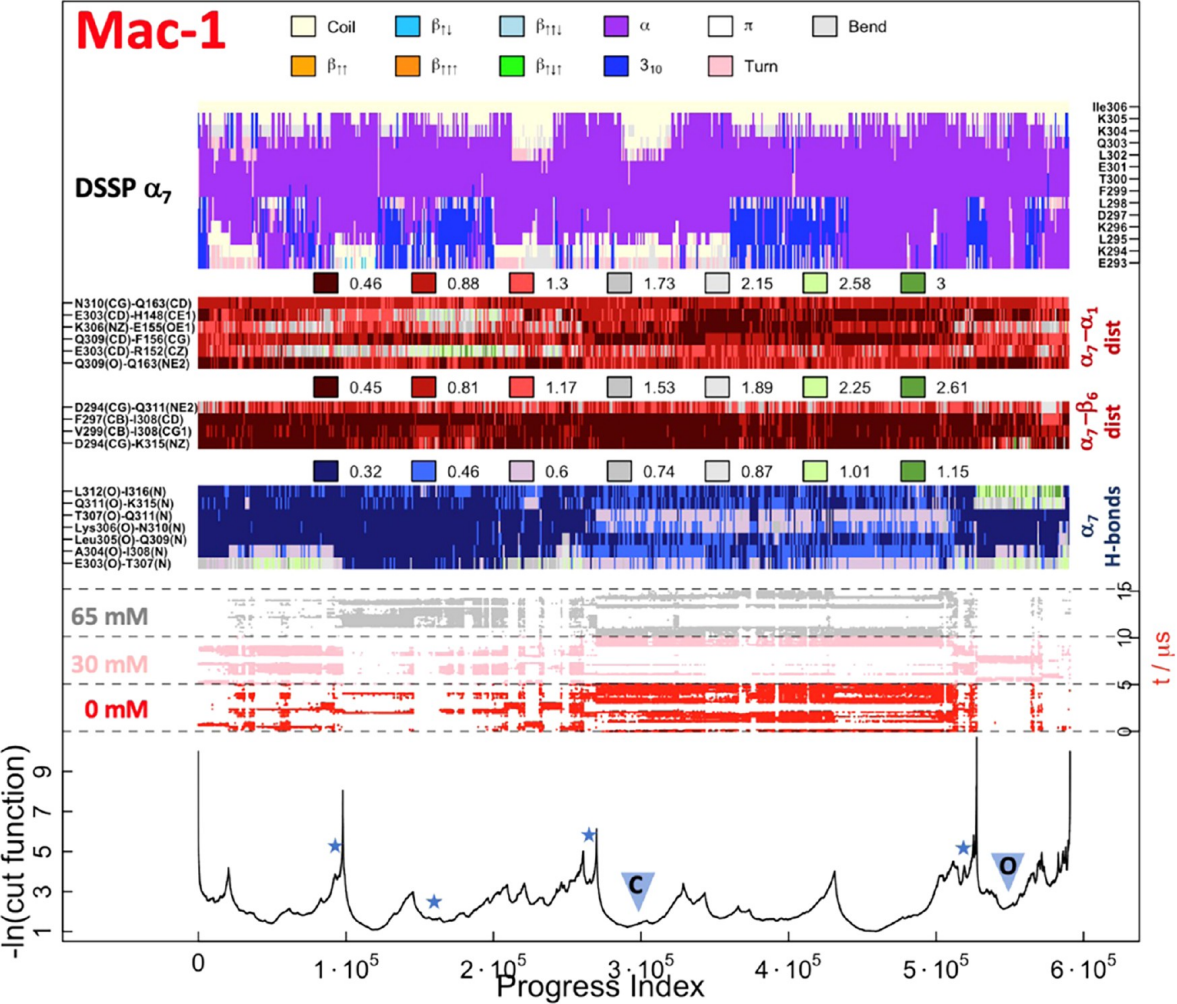
SAPPHIRE plot for the conformational space of the Mac-1 β_6_–α_7_ pocket. The caption is the same as for Figure 6.

**Figure 9:**
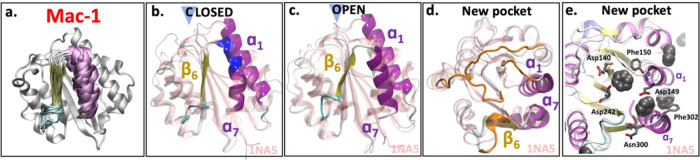
Simulation snapshots of Mac-1. (a) Structural overlap of 60 snapshots extracted from the simulations highlighting the dynamics of α_7_. (b) Representative snapshots of the closed and (c) open conformations of the allosteric pocket extracted from the center of the basins highlighted in SAPPHIRE plot (Figure 8). The structural overlap to the closed Mac-1 crystal structure (PDB ID: 1na5(30)) is shown. Highlighted are β_6_, α_7_, and α_1_. (Right) Top view of the new pocket. (d, e) Top view of the new pocket, highlighting the loops (orange loops in (d)) and the insertion of benzene molecules (shown in van der Waals representation in (e)).

**Figure 10:**
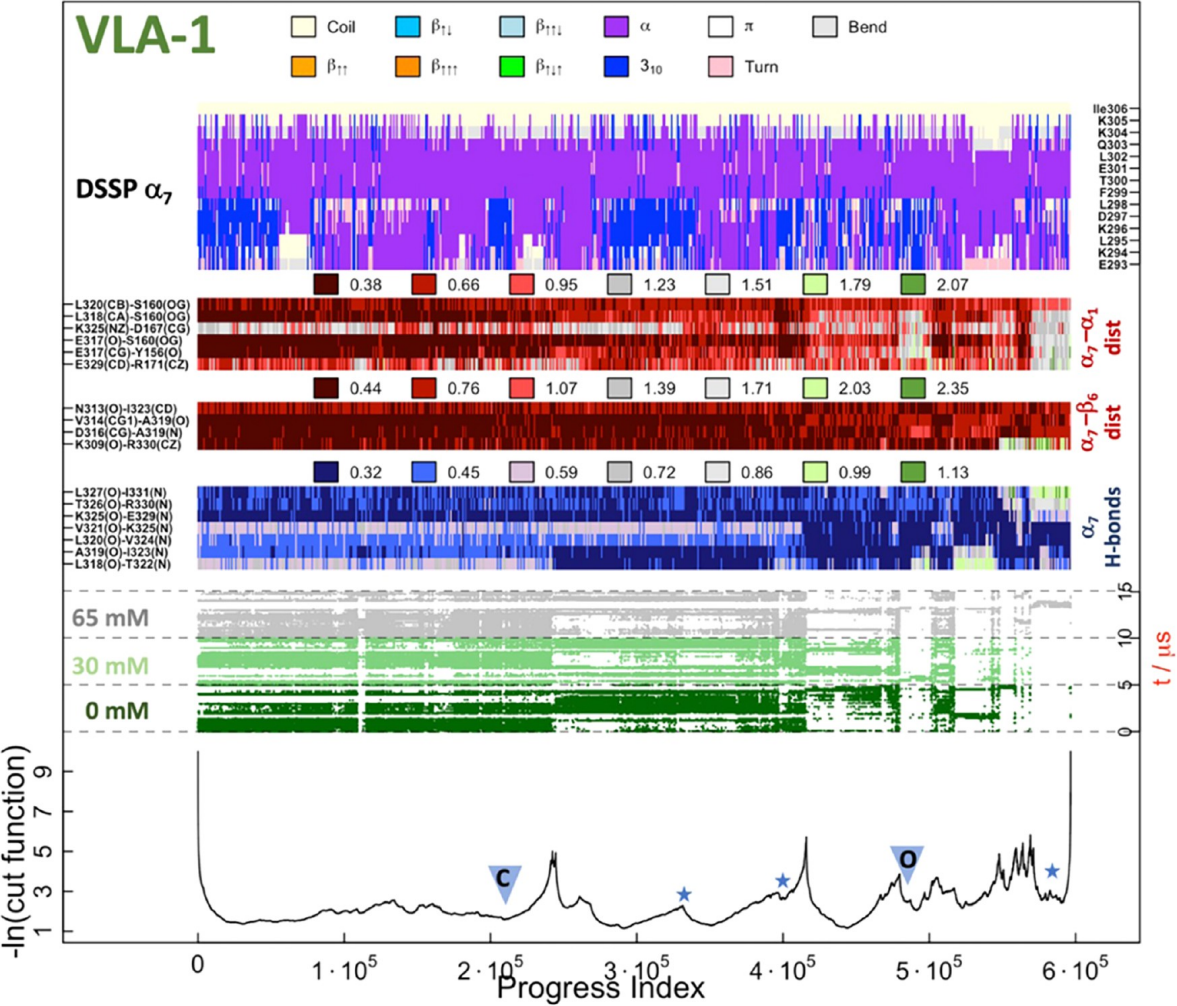
SAPPHIRE plot for the conformational space of the VLA-1 β_6_–α_7_ pocket. The caption is the same as for Figure 6.

**Figure 11:**
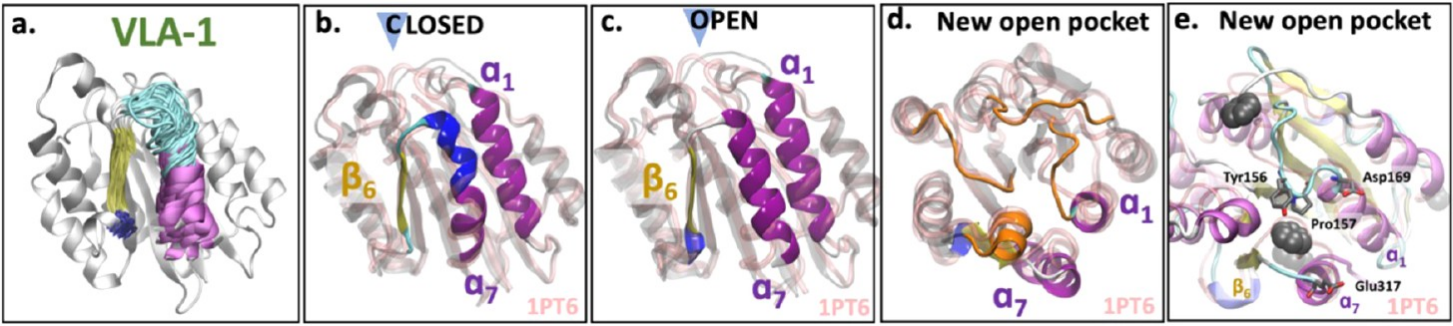
Simulation snapshots of VLA-1. (a) Structural overlap of 60 snapshots extracted from the simulations highlighting the dynamics of α_7_. (b) Representative snapshots of the closed and (c) open conformations of the allosteric pocket extracted from the center of the basins highlighted in SAPPHIRE plot (Figure 10). The structural overlap to the closed VLA-1 crystal structure (PDB ID: 1pt6(31)) is shown. Highlighted are β_6_, α_7_, and α_1_. (d, e) Top view of the new pocket, highlighting the loops (orange loops in (d)) and the insertion of benzene molecules (shown in van der Waals representation in (e)).

To gain structural insight into the conformational modifications induced by the organic solvent, we employ SAPPHIRE (States and Pathways Projected with High Resolution) analysis.^35,36^ The SAPPHIRE analysis (Figures 6–11) gives an overview of the cumulative sampling for LFA-1, Mac-1, and VLA-1, respectively, at 300 K. In brief, the idea consists of reordering all trajectory snapshots based on geometric similarity as defined by a metric given as input (see Tables S1–S3 for the full definition of the metric). The data is partitioned into basins consisting of similar snapshots without any a priori clustering or overlap between the distinct states. The resulting sequence of snapshots is referred to as the progress index. The cut functions (black lines in the lower part of the plots) represent pseudo-free energy profiles that separate the individual states and help identify the barriers between different basins. The progress index is annotated with suitable variables, which highlight the conformationally and/or kinetically homogeneous states and the dynamics between them. The dot patterns (on the *y* axis) represent the actual time of occurrence of the reordered snapshots; the three different concentrations are separated by horizontal dashed lines and highlighted by the different colors.

The SAPPHIRE plots^35,36^ are annotated with a set of interatomic distances, which contribute to the integrity of the allosteric pocket, i.e., donor–acceptor α_7_ hydrogen-bond distances α_7_ H-bonds, α_7_–β_6_ distances (α_7_–β_6_ dist), and α_7_–α_1_ distances (α_7_–α_1_ dist), and the secondary structure assignment of α_7_ (top annotation). The barriers in the cut function (i.e., local maxima on the profile in black at the bottom of Figures 6, 8, and 10) separate individual metastable states, whose weights are quantifiable by the progress-index segment between two consecutive barriers. Importantly, recurrence across the individual simulations and systems shows that most basins are sampled several times and in different simulation systems (dark blue, light blue, and green dots above the cut function).

The SAPPHIRE analysis finds multiple metastable states of the LFA-1 α I allosteric pocket. As the interest of this study is to identify and characterize the open conformation of the pocket, the following analysis will focus mainly on the states that describe this arrangement and not address in detail every basin. The most recurrent state across all simulations, but predominantly in the systems devoid of benzene molecules, is the closed state, in which no potential ligand can access the α I allosteric pocket. For instance, the basin between 1.8 × 10^5^ and 2.3 × 10^5^ highlights states characterized by the presence of the E284-K305 and K287-E301 salt bridges both of which stabilize the closed conformation of the LFA-1 allosteric pocket (Figure 6, top center). In contrast, the following basin (between 2.3 × 10^5^ and 2.7 × 10^5^) highlights states that are primarily identified in the systems with benzene molecules and only minimally present in the simulations devoid of fragment probes. Furthermore, none of the above-mentioned salt bridges are formed and the structural overlap to the crystal structure (PDB ID: 2ica(5)) reveals an open druggable conformation of the α I allosteric pocket (Figure 6, bottom right). In the open state the K287-E284 backbone contact is formed, an arrangement that is typical for the LFA-1 α I domain/inhibitor structures.^5^ Further open conformations are marked along the progress index by the blue stars. Importantly, throughout the simulations and independently of the addition of benzene molecules, the integrity of the helical content in α_7_ is largely preserved. Additionally, most other states are not fully closed but rather intermediate (quasi-open) configurations between the open and closed conformations and do not allow the access of a potential inhibitor. During the simulations a second potentially druggable pocket is identified at the β_6_–α7 loop and the C-terminus of α_1_ of the LFA-1 α I domain. This β_6_–α_7_–α_1_ pocket forms because of the rearrangement of the loops at the interface and of the two helices with respect to each other (Figure 7, Movie S7). It is identified independently of the conformation of the α I allosteric pocket and across all simulation systems.

In contrast to LFA-1, the identification of open allosteric pockets in Mac-1 and VLA-1 is a more challenging task. The SAPPHIRE analysis of Mac-1 reveals that the allosteric β_6_–α_7_ pocket is predominantly closed, independently of the benzene concentration (Figure 8). This is consistent with the distance analysis in Figure 4a, which indicates the long lifetime of the D294-K315 salt bridge. We identify a limited number of basins, in which open conformations of the β_6_–α_7_ pocket are sampled (Figure 9 and Movie S8). These are present in the systems with benzene molecules (e.g., between 5.2 × 10^5^ and 5.8 × 10^5^ and marked by stars in Figure 8) and are characterized by the breakage of the D294-K315 salt bridge, which is induced by the insertion of benzene molecules between α_7_ and β_6_ (Movie S8). Rarely, the quasi-opening of the pocket is owed to the unwinding of the C-terminus of α_7_, which leads to a loss in the secondary structure. Like LFA-1, the opening of the new pocket coincides with the rearrangement of the loops at the β_6_–α_7_–α_1_ interface, which arises due to the frequent interactions of the benzene molecules with the α_7_–β_6_ loop (specifically residues N300 and F302, see Figure 4c, Figure 9e), the N-terminus of α_1_ (residues D149 and F150, see Figure 9e) and residues D140 and D242 in the surrounding loops (Figure 9e). Consequently, the E303-R152 salt bridge linking α_7_ to α_1_ is broken, the helices are pushed away from each other, and the β_6_–α_7_–α_1_ pocket forms (Figure 9, e.g., the basin between 1.5 × 10^5^ and 2 × 10^5^ and Movie S8).

In VLA-1, the SAPPHIRE analysis discriminates less between β_6_–α_7_ open and closed conformations as most states are populated across most basins (Figure 10). In fact, the K309-R330 contact remains populated over 70% across the simulations independently of the benzene concentration (Figure 4a) and dissociates in one of the simulations at high benzene concentrations (last basin in Figure 10 and Movie S9).

Aside from this, the open conformation is short-lived and sampled only in small basins (marked along the progress index by stars). On the other hand, like in Mac-1, the β_6_–α_7_–α_1_ pocket is more prone to open through loop and concurrent helix rearrangements at the interface (Figure 11d,e and Movie S9). The displacement of the α_7_ helix is achieved through the intercalation of benzene molecules between α_1_ and α_7_, facilitated by frequent interactions of benzene molecules with residues Y156, P157, and E317 (Figure 11e). The benzene molecules trigger a rigid body motion of the N-terminus of α_7_ (captured also in Figure 3 by the increased flexibility of the N-terminus on the short timescale), which leads to a larger separation between residues E317 and Y156 and implicitly a helix rearrangement. Additionally, benzene intercalation between the α_1_ and α_7_ helices stimulates at times the parallel downward sliding of α_7_. Interestingly, this conformational change has been previously induced in LFA-1 by introducing artificial disulfide-bond ratchets. Moreover, this artificial pulling down of the α_7_ helix led to a progressive increase in α I domain binding affinity.^37^

### Potential Druggability of the β_6_–α_7_ and β_6_–α_7_–α_1_ Pockets

**Figure 12:**
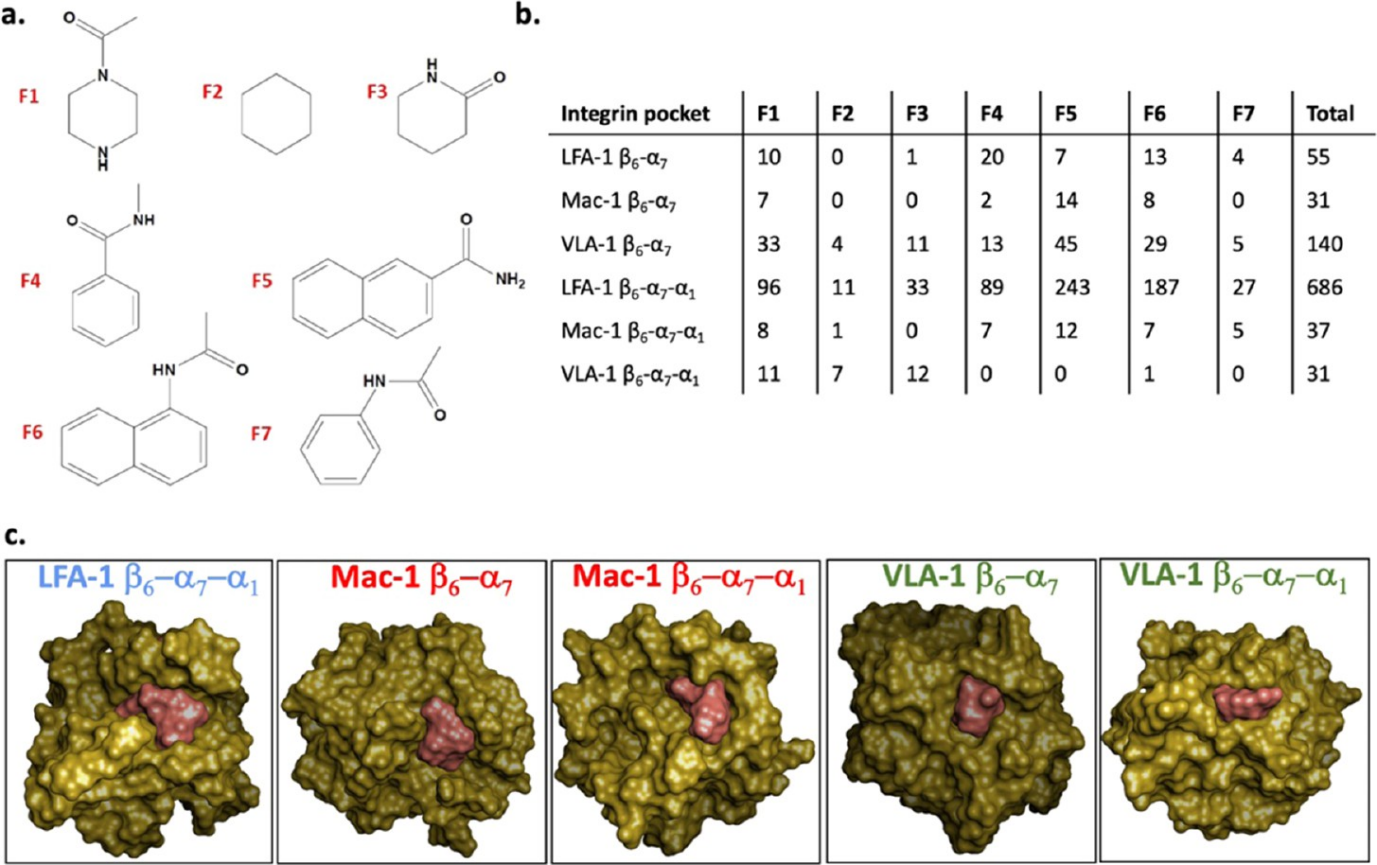
Druggability assessment of the β_6_–α_7_ and β_6_–α_7_–α_1_ pockets. (a) Chemical structures of the fragments used for SEED docking analysis (F1: 1-acetylpiperidine, F2: cyclohexane, F3: 2-piperidon, F4: *N*-methylbenzamide, F5: 2-naphthoic acid amide, F6: *N*-(1-naphthyl)-acetamide, F7: *N*-phenylacetamide). (b) Fragments were docked into the α I domain β_6_–α_7_ and β_6_–α_7_–α_1_ pockets of the integrins LFA-1, Mac-1, and VLA-1, respectively, using the SEED program. SEED calculations were performed as described in the Methods section. The number of fragment poses with *E* < −12 kcal/mol detected by SEED are shown. (c) Surface representation of the α I domains (gold) and the combined surface for all docked fragments with energy below −12 kcal/mol (salmon). For the β_6_–α_7_–α_1_ pocket in LFA-1, the energetic cutoff was set at −13 kcal/mol. The α I domains used are representative snapshots for open conformations selected from the SAPPHIRE analysis, i.e., from the minimum of the basins highlighted in Figures 6, 8, and 10.

To assess the druggability of the newly discovered pockets, i.e., the β_6_–α_7_ pockets in Mac-1 and VLA-1, and the β_6_–α_7_–α_1_ pockets in all three α I domains, small rigid fragments were docked into the sites using the open source program SEED.^38^ The fragments are mainly hydrophobic yet contain an amide extension as a possible linker for the addition of hydrophilic groups, with the exception of cyclohexane (Figure 12a). We provide a first overview of the binding sites, as the *de novo* small-molecule design is beyond the scope of the current study.

Five open conformations are selected from the SAPPHIRE analysis based on the size of the pocket (Figure 12). For comparison, SEED docking was also applied to the LFA-1 crystal structure, used as a starting conformation for the MD simulations. In the preparation of the docking process, the structures are energy-minimized with restrains applied on the C_α_-atoms. The number of fragment poses with a SEED energy of less than −12 kcal/mol is summarized in Figure 12b. Further, to visualize the size of each pocket available for drug design, the combined surfaces of fragment poses at the energy cutoff are shown together with the protein surfaces (Figure 12c). Only for the LFA-1 β_6_–α_7_–α_1_ site a cutoff of −13 kcal/mol was used for the combined fragment surface (Figure 12c). As expected, the fragments interact with the pockets through their hydrophobic part. In most cases, the amide linker moieties of the fragments point toward the outside of the pocket. These positions of the linker moieties may allow the attachment of solubilizing groups or other groups which could improve the druggability of potential ligands.

Moreover, the results indicate that the β_6_–α_7_ pocket of VLA-1 is larger than the analogous pocket in the LFA-1 crystal structure. However, it needs to be taken into account that the LFA-1 pocket has adapted its size to the ligand present in the crystal structure. Thus, it could possibly provide space for larger ligands. The β_6_–α_7_ pocket in Mac-1 is the smallest, but could still provide enough space to bind an organic molecule. Further, in the case of the β_6_–α_7_–α_1_ pocket, the one in LFA-1 is clearly the largest relative to those detected in Mac-1 and VLA-1. The LFA-1 β_6_–α_7_–α_1_ pocket has the shape of an open tunnel that traverses the whole protein. In comparison, the β_6_–α_7_–α_1_ pocket of Mac-1 is rather broad but not very deep such that bigger fragments do not achieve the fit as observed for fragments binding to the LFA-1 pocket. The VLA-1 β_6_–α_7_–α_1_ pocket is the smallest but is nevertheless suited to provide enough space for a ligand (Figure 12c). Whether ligands binding to the newly identified sites in the α I domains will have similar effects on the whole integrin and indeed modulate its function remains to be tested.

## Discussion

Allosteric modulation of receptors creates opportunities for drug discovery and development which are potentially superior to classic orthosteric modulation. Specifically, allosteric modulation avoids the risk of inadvertently eliciting effects naturally triggered by ligand binding to the orthosteric site.^39,40^ Integrins represent a family of therapeutically relevant receptors which are of particular interest for allosteric rather than orthosteric modulation. This is because pharmacologic interference with the orthosteric ligand binding site is known already to have the potential of inducing major unwanted effects, such as unintended agonist-like effects of integrin inhibitors. A first prototypic example establishing that allosteric modulation can be applied to members of the integrin family exists with the integrin LFA-1. Small molecules targeting the α I allosteric pocket of LFA-1 have been shown to stabilize LFA-1 in a distinct conformational state, translating into inhibition^14^ or, observed less frequently, into activation of LFA-1 function.^41^

Despite this promise, no rigorous attempts have been made to date towards identifying molecules targeting putative α I allosteric sites similar to the LFA-1 α I allosteric site in other integrins. A few in silico studies provide evidence that such sites may exist in integrins closely related to LFA-1, e.g., αxβ_2_ (CD11c/CD18)^42^ and Mac-1.^43−45^ In addition, there is evidence suggesting that the putative α I allosteric site of Mac-1 can accommodate inhibitors^44^ as well as activators^43,45^ of Mac-1 function. However, these investigations did not prompt further research into the accessibility and druggability of putative α I allosteric pockets in other integrins. One important reason for this lack of progress is the difficulty in identifying therapeutically tractable allosteric pockets.^24,25^ It is worth noting that the LFA-1 α I allosteric pocket itself was discovered by serendipity rather than by prospectively defined research. Moreover, our results show that the LFA-1 α I allosteric pocket closes promptly in the absence of ligands, i.e., can be missed easily unless searched for specifically, underlining the difficulty of uncovering hidden allosteric sites. In fact, all LFA-1 α I domain crystal structures published to date are ligand-bound and capture the *open* conformation of the α I allosteric site, further suggesting that the *open* configuration may be unstable in the absence of ligands and hence difficult to access.^32,33^

By applying mixed-solvent MD simulations to integrins, the current study demonstrates and characterizes the potential opening of cryptic α I allosteric sites of different integrins. We investigated the concentration-dependent effects of benzene molecules on the α I domain conformational state of three therapeutically relevant integrins, i.e., LFA-1 (as a control), Mac-1, and VLA-1. Our results show that the putative α I allosteric sites of Mac-1 and VLA-1 are predominantly closed under physiological conditions yet are susceptible to open at the β_6_–α_7_ interface (region analogous to the α I allosteric site of LFA-1) in benzene-rich solvent. Unlike LFA-1, the *closed* conformations in Mac-1 and VLA-1 are stabilized mainly by hydrophobic contacts between β_6_ and α_7_. By enriching the solvent with benzene molecules, the populations of stabilizing contacts decrease, which in turn leads to the opening or quasi-opening of the α I domain pockets of Mac-1 and VLA-1, respectively. Our results are in line with previous findings showing that the salt bridge interaction of α_7_ with other parts of the α I domain differs for distinct members of the β_2_ integrin subfamily [i.e., LFA-1, Mac-1, and αxβ_2_] and is associated with diverse α_7_ flexibility.^34^

A limitation intrinsic to the cryptic site opening approach described here is that the degree of site opening is dependent on the chemical nature of the fragment probe selected.^25^ In the current study we use benzene (rather than alternative fragments such as pyridine, imidazole, indole, or pyrimidine) for several reasons. First, the molecular structure of benzene mimics lovastatin, the first inhibitor described to bind to the α I allosteric pocket of LFA-1^12,13^ Second, benzene is the most common hydrophobic ring fragment present in approved drugs.^46^ Thirdly, aromatic rings are constitutive to broad spectra of natural biomolecules, including molecules of potent immunomodulatory capacity.

Mechanistically three different modes of action of the α I allosteric pocket opening are to be considered, i.e., conformational selection, induced-fit, or a mixed mechanism.^25^ In the first case, a cryptic pocket opens transiently, yet spontaneously, and is then occupied by the ligand. In the second case, the pocket does not open spontaneously and needs the help of ligand for opening in an induced-fit style. The mixed mechanism is characterized by a slight spontaneous opening, which requires ligand to reach the fully opened state.^25^ In our mixed-solvent MD simulations we observe the occasional openings of the α I allosteric pocket of LFA-1 in water. In the presence of solvent/benzene mixtures, the intercalation of benzene molecules between the β_6_ and α_7_ interface is observed, enhancing pocket opening. This result indicates that the α I allosteric site of LFA-1 opens according to the mixed mechanism model. In contrast, Mac-1 and VLA-1 appear to require benzene for the opening of their putative α I allosteric pockets (β_6_–α_7_ pocket), thus following the induced-fit model.

We project that the Mac-1 and VLA-1 β_6_–α_7_ pockets can be utilized to design small-molecule modulators of these two integrins, as has been accomplished successfully in the past with the analogous site within LFA-1.^14^ Indeed, SEED analysis of the respective integrin α I domains revealed that allosteric modulation by small molecules may be possible through these pockets. For Mac-1, this finding is in agreement with earlier molecular docking studies referred to above.^43−45^ For VLA-1 the current study is the first study to suggest that this integrin may be targetable via the β_6_–α_7_ pocket. Although VLA-1 is a promising therapeutic target,^27^ pharmacological progress in designing VLA-1 inhibitors has been slow, to date. To the best of our knowledge SAN-300, a humanized monoclonal antibody apparently no longer in development, was the sole modality explored clinically for the treatment of rheumatoid arthritis.^27^ As anti-integrin antibodies are known to be associated with substantive limitations,^18^ allosteric targeting of VLA-1 by small molecules may emerge as an important translational advance. Target indications of high medical need under consideration for future VLA-1 inhibitors include inflammatory, fibrotic, and malignant diseases.^47−49^

Unexpectedly, our results show the formation of a second potentially druggable pocket (termed here β_6_–α_7_–α_1_ pocket), located at the interface between the β_6_–α_7_ loop and the N-terminus of α_1_ in all three α I domains. This novel pocket opens independently of the β_6_–α_7_ conformation. It forms through the concomitant motion of the helices, driven by the intercalation of benzene molecules between α_1_ and α_7_ and stimulated by frequent interactions with aromatic residues. Earlier simulations of the α I domains of several integrins found that the β_6_–α_7_ loop of the β_6_–α_7_–α_1_ pocket can adopt closed, open or intermediate conformations, with the first being observed more frequently in VLA-1 than in LFA-1 and Mac-1.^50^ The authors attribute the stability of the *closed* conformation to the R152-E303 salt bridge connecting α_1_ to α_7_ in Mac-1. Our results are in line with this hypothesis. We additionally show that the breakage of the salt bridge can be induced and occurs as a consequence of the intercalation of benzene molecules between α_1_ and α_7_, thereby opening the β_6_–α_7_–α_1_ allosteric pocket. In VLA-1, the interaction between α_1_ and α_7_ is mainly hydrophobic with no salt bridge stabilizing the interfacial contact between the β_6_–α_7_ loop and α_1_. Intriguingly, our SEED analysis provides the first evidence that molecules interacting with the novel β_6_–α_7_–α_1_ pocket of the integrin α I domains can be designed, as also shown for the newly identified β_6_–α_7_ pockets of Mac-1 and VLA-1. However, it remains to be explored whether molecules targeting these newly identified allosteric pockets will exhibit effects similar to the small-molecule modulators which bind to the classical α I allosteric site of LFA-1. Moreover, further structural characterization of the newly described β_6_–α_7_ and β_6_–α_7_–α_1_ pockets in integrin α I domains, e.g., crystallization of the respective α I domains with benzene and/or other ligands identified by molecular docking, are required for verification.

In conclusion, by applying mixed-solvent MD simulations to integrins, we demonstrate that pockets similar to the α I allosteric pocket of the integrin LFA-1 can be rendered accessible by adjusting solvent conditions. For the integrins Mac-1 and VLA-1, specifically, we show that compounds interacting with their putative α I allosteric sites can be identified readily using molecular docking. In addition, we identify a novel potentially druggable cryptic pocket at the β_6_–α_7_–α_1_ interface. Taken together, our findings indicate that integrin allosteric site hopping might be possible and might become a strategy for extending the discovery of next-generation α I allosteric integrin modulators. Specifically, it appears conceivable that the mixed-solvent MD simulation approach pioneered for three integrins in the current study may become extended to systematically assess the allosteric targetability of 9 of 24 integrins carrying α I domains. As several of these integrins, including the three integrins analyzed here, play decisive roles in diseases for which there is no cure, to date, substantive therapeutic benefit may derive from these future investigations.

## Methods

### System Preparation

The crystal structures of LFA-1 (PDB ID: 2ica(5)), Mac-1 (PDB ID: 1na5(30)), and VLA-1 (PDB ID: 1pt6(31)) were used as starting points for the molecular dynamics simulations. For consistency across the three proteins, Mac-1 and VLA-1 were cut beyond I316 and I331, respectively.

### Simulation Protocol

The simulations were carried out using the GROMACS 2020.5 simulation package.^51^ All simulations were performed using the all-atom CHARMM36m force field^52^ and the TIP3P water model.^53^ The N- and C-termini of the proteins were uncapped. Each complex was first solvated in a cubic box (edge length of 8 nm) with TIP3P water molecules to which 150 mM NaCl were added, including neutralizing counterions. Additionally, in the simulations in an organic solvent, benzene molecules were randomly distributed in the box at noninteracting distances from the protein. Following the steepest descent minimization, the systems were first equilibrated under constant pressure for 5 ns. The temperature and pressure were maintained constant at 300 K and 1 atm, respectively, by using the modified Berendsen thermostat (0.1 ps coupling^54^) and barostat (2 ps coupling^55^). The production simulations were performed in the NVT ensemble in the absence of restraints. The short-range interactions were cut off beyond a distance of 1.2 nm, and the potential smoothly decays to zero using the Verlet cutoff scheme. Periodic boundary conditions were used and the Particle Mesh Ewald (PME) technique^56^ was employed (cubic interpolation order, real space cutoff of 1.2 nm, and grid spacing of 0.16 nm) to compute the long-range electrostatic interactions. Distances of covalent bonds involving H atoms were constrained by means of a fourth-order LINCS algorithm with 2 iterations.^57^ Six different sets of simulations were carried out for each system, at three concentrations of benzene (0, 30, and 65 mM) and temperature values of 300 and 350 K. Five independent 1-μs simulations were carried out for each set for a total cumulative sampling of 30 μs. In all simulations the time-step was fixed to 2 fs and the snapshots were saved every 25 ps. The higher temperature is employed to enhance the sampling; the density of water is kept at the value of the 300 K simulations (i.e., same volume of the box and same number of water molecules) to perturb the free energy surface as little as possible.^58−60^ The benzene concentrations were chosen such that the proteins are not denatured and that no aggregation of benzene molecules is observed in bulk.^61^ Nine movies resulting from this work are shown in the Supporting Information.

### SEED Analysis

SEED calculations were carried out with version 3.3.6 of the program^38,62^ (https://gitlab.com/CaflischLab/SEED). The force field-based energy function used in SEED consists of four terms, namely, electrostatic and van der Waals interactions between the protein and the fragment, and the electrostatic desolvation penalty of the protein and the fragment. CHARMM36m partial charges and van der Waals parameters were assigned to proteins and fragments using the molecular graphics program Wit!P (http://www.biochem-caflisch.uzh.ch/download). For all calculations, dielectric constants of 2.0 and 78.5 were used for solute and solvent, respectively. All polar fragments were placed according to the option “both”, i.e., in optimal polar and apolar regions. Pose clustering was carried out individually for each fragment, and for each cluster, only the member with the most favorable SEED total energy was saved.

## Data Availability

PDB files were downloaded from the RCSB Protein Data Bank (https://www.rcsb.org). GROMACS (https://www.gromacs.org/) was used to perform the molecular dynamics simulations and SEED for the docking step (https://gitlab.com/CaflischLab/SEED). Campari (https://campari.sourceforge.net) was used for analyses. Input files and analysis scripts are available at https://github.com/ilieim/DecryptingIntegrins.

## Supporting Information Available

RMSD analysis of the MD simulations at 350 K and set of interatomic distances used for the SAPPHIRE-based clustering (PDF)
LFA-1, 0 mM benzene (MP4)
Mac-1, 0 mM benzene (MP4)
VLA-1, 0 mM benzene (MP4)
LFA-1, 30 mM benzene (MP4)
Mac-1, 30 mM benzene (MP4)
VLA-1, 30 mM benzene (MP4)
LFA-1, 65 mM benzene (MP4)
Mac-1, 65 mM benzene (MP4)
VLA-1, 65 mM benzene (MP4)
