# Supplementary material for: Decrypting Integrins by Mixed-Solvent Molecular Dynamics Simulations
Source: J Chem Inf Model. 2023 Jun 13;63(12):3878–91. doi: 10.1021/acs.jcim.3c00480 (PMC10302478; doi:10.1021/acs.jcim.3c00480)
Supplement: Supplementary file 1 — ci3c00480_si_001.pdf [file ci3c00480_si_001.pdf]

Supporting Information:
Decrypting integrins by mixed-solvent molecular dynamics simulations

Ioana M. Ilie[1,2*], Claus Ehrhardt[3], Amedeo Caflisch[3] and Gabriele Weitz-Schmidt[4*]

[1]van't Hoff Institute for Molecular Sciences, University of Amsterdam, P.O. Box 94157, 1090 GD Amsterdam, The Netherlands
[2]Amsterdam Center for Multiscale Modeling (ACMM), University of Amsterdam, P.O. Box 94157, 1090 GD Amsterdam, The Netherlands
[3]Department of Biochemistry, University of Zurich, Winterthurerstrasse 190, CH-8057 Zurich, Switzerland
[4]AlloCyte Pharmaceuticals AG, Hochbergerstrasse 60C, CH-4057 Basel, Switzerland

*corresponding authors: i.m.ilie@uva.nl; gabriele.weitz@allocyte-pharma.com

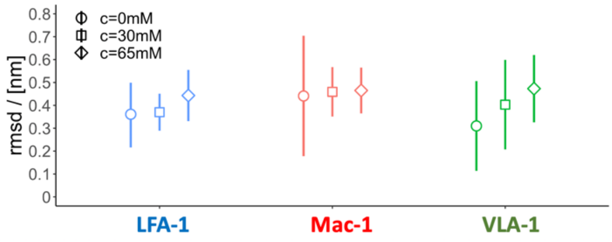

Figure S1 Average root mean-square-deviations (RMSD) of α_7 with respect to the rest of the I domain at 350K. The reference structure is the closed conformation. For LFA-1 the reference structure is a representative closed conformation post-equilibration. First structural alignment of the individual snapshots saved along the MD simulations is carried out on the Ca-atoms of all residues except those comprised in α_7, i.e. F292-I306, F302-I316 and E317-I331 for LFA-1, Mac-1 and VLA-1, respectively. Then for each MD snapshot the α_7 Ca-RMSD is calculated as $\sqrt{\frac{1}{N}\sum_{i=1}^{N}\left(\boldsymbol{r}_i - \boldsymbol{r}_i^{ref}\right)^2}$, where $\boldsymbol{r}_i$ and $\boldsymbol{r}_i^{ref}$ are the actual and reference coordinates, respectively, of the α_7 Ca atom i and N the number of residues in α_7. The error bars represent the standard error of the mean calculated as the standard deviation of the average values over the five independent runs.

Table S1 Set of interatomic distances used for the LFA-1 SAPPHIRE based clustering.

E293 backbone O	Backbone N atoms of D297, F299, E301, Q303, K305
K294 backbone O	Backbone N atoms of L298, T300, L302, K304, I306
L295 backbone O	Backbone N atoms of F299, E301, Q303, K305
K296 backbone O	Backbone N atoms of T300, L302, K304, I306
D297 backbone O	Backbone N atoms of E301, Q303, K305
L298 backbone O	Backbone N atoms of L302, K304, I306
F299 backbone O	Backbone N atoms of Q303, K305
T300 backbone O	Backbone N atoms of K304, I306
E301 backbone O	Backbone N atom of K305
L302 backbone O	Backbone N atom of I306
D290 sidechain CG	K294 sidechain NZ
T291 backbone O	K294 backbone N
L289 sidechain CG	K294 backbone O
I288 backbone O	L298 sidechain CG
K287 sidechain NZ	E301 sidechain CD
E284 sidechain CD	K305 sidechain NZ
F292 sidechain CG	E146 sidechain CD
E293 sidechain CD	K149 sidechain Nz
K296 sidechain NZ	D152 sidechain CG
K296 sidechain NZ	D156 sidechain CG

F299 sidechain CG	F153 sidechain CG
Q303 sidechain CD	L161 sidechain CG
Q303 sidechain OE1	K160 sidechain NZ

Table S2 Set of interatomic distances used for the Mac-1 SAPPHIRE based clustring.

E303 backbone O	Backbone N atoms of T307, Q309, Q311, R313, K315
A304 backbone O	Backbone N atoms of I308, N310, L312, E314, I316
L305 backbone O	Backbone N atoms of Q309, Q311, R313, K315
K306 backbone O	Backbone N atoms of N310, L312, E314, I316
T307 backbone O	Backbone N atoms of Q311, R313, K315
I308 backbone O	Backbone N atoms of L312, E314, I316
Q309 backbone O	Backbone N atoms of R313, K315
N310 backbone O	Backbone N atoms of E314, I316
Q311 backbone O	Backbone N atom of K315
L312 backbone O	Backbone N atom of I316
D294 sidechain CG	K315 sidechain NZ
N301 sidechain OD1	E303 backbone N
N301 backbone O	A304 backbone N
V299 backbone CB	I308 sidechain CG1
F297 backbone CB	I308 sidechain CD
D294 sidechain CG	Q311sidechain NE2
Q309 backbone O	Q163 sidechain NE2
E303 sidechain CD	R152 sidechain CZ
Q309 sidechain CD	F156 sidechain CG
K306 sidechain NZ	E155 sidechain OE1
E303 sidechain CD	H148 sidechain CE1
N310 sidechain CG	Q163 sidechain CD

Table S3 Set of interatomic distances used for the VLA-1 SAPPHIRE based clustering.

L318 backbone O	Backbone N atoms of T322, V324, T326, G328, R330
A319 backbone O	Backbone N atoms of I323, K325, L327, E329, I331
L320 backbone O	Backbone N atoms of V324, T326, G328, R330
V321 backbone O	Backbone N atoms of K325, L327, E329, I331
T322 backbone O	Backbone N atoms of T326, G328, R330
I323 backbone O	Backbone N atoms of L327, E329, I331
V324 backbone O	Backbone N atoms of G328, R330
K325 backbone O	Backbone N atoms of E329, I331
T326 backbone O	Backbone N atom of R330
L327 backbone O	Backbone N atom of I331
K309 backbone O	R330 sidechain CZ
D316 sidechain CG	A319 backbone N
V314 sidechain CG1	A319 backbone O
N313 backbone O	I323 sidechain CD
F312 sidechain CG	L327 sidechain CD1
K309 backbone O	R330 sidechain CG
E329 sidechain CD	R171 sidechain CZ
E317 sidechain CG	Y156 backbone O
E317 backbone O	S160 sidechain OG
K325 sidechain NZ	D167 sidechain CG
L318 backbone CA	S160 sidechain OG
L320 backbone CB	S160 sidechain OG

Molecular dynamics simulation movies

The movies show the α I domains of the three studied systems on a timescale of 1 μs, with snapshots captured every 1 ns. The α I domain is shown in cartoon representation using secondary structure coloring, *i.e.* purple for α helices blue for 3_{10} helices, yellow for β-sheets, white and cyan for loops and coils, respectively. The α_7 helix is highlighted. The *closed* conformation is shown in white cartoon representation. For LFA-1, the closed conformation was selected post-equilibration. For Mac-1 and VLA-1, the *closed* conformations correspond to the crystal structures. Benzene molecules are shown as grey spheres.

S1. LFA-1, 0 mM benzene

In aqueous solution, the α I domain of LFA-1 shows small deviations from the *closed* conformation.

S2. Mac-1, 0 mM benzene

In aqueous solution, the α I domain of Mac-1 shows small deviations from the *closed* conformation.

S3. VLA-1, 0 mM benzene

In aqueous solution, the α I domain of VLA-1 shows small deviations from the *closed* conformation.

S4. LFA-1, 30 mM benzene

The addition of benzene molecules stimulates the opening of the β_6-α_7 pocket. At first, single benzene molecules interact with the β_6-α_7 pocket without disturbing its *closed* conformation. The helix moves then away from β_6 as more benzene molecules intercalate between β_6 and α_7. α_7 moves back towards β_6, thereby closing the pocket as the benzene molecules leave the pocket. Occasionally, benzenes intercalate between α_7 and α_1 pushing the two helices away from each other.

S5. Mac-1, 30 mM benzene

Benzene molecules interact with residues in the β_6-α_7-α_1 pocket, thereby inducing the opening of the new pocket. Seldom benzene interaction with the β_6-α_7 pocket does not suffice to generate the opening of the allosteric pocket.

S6. VLA-1, 30 mM benzene

Benzene molecules intercalate in the β_6-α_7 pocket and occasionally between α_7 and α_1, inducing sporadic shifts of α_7 from the *closed* structure.

S7. LFA-1, 65 mM benzene

Multiple benzene molecules are inserted into the β_6-α_7 pocket, which prompt the wide opening of the allosteric site. Additionally, the new β_6-α_7-α_1 pocket forms due to the insertion of benzene molecules.

S8. Mac-1, 65 mM benzene

Benzene molecules prompt the opening of both pockets through the shifting of the α_7 helix.

S9. *VLA-1, 65 mM benzene*

Benzenes intercalate between helices α_7 and α_1, prompting the shift of the α_7 helix.
